# Characterization of the Normal Portal and Hepatic Blood Flow of Adult Holstein-Friesian Cows

**DOI:** 10.3390/ani9060386

**Published:** 2019-06-22

**Authors:** J. Daniel Barreiro-Vázquez, Marta Miranda, M. Isabel Barreiro-Vilanova, F. Javier Diéguez, Andrés Barreiro-Lois

**Affiliations:** 1 Department of Anatomy, Animal Production and Clinical Veterinary Sciences, Faculty of Veterinary Medicine, Universidade de Santiago de Compostela, 27002 Lugo, Spain; josedaniel.barreiro@usc.es (J.D.B.-V.); franciscojavier.dieguez@usc.es (F.J.D.); andres.barreiro@usc.es (A.B.-L.); 2 Veterinary Teaching Hospital “Rof-Codina”, Faculty of Veterinary Medicine, Universidade de Santiago de Compostela, 27002 Lugo, Spain; 3 Department of Physiology, Faculty of Veterinary Medicine, Universidade de Santiago de Compostela, 27002 Lugo, Spain; mariaisabel.barreiro@usc.es

**Keywords:** Doppler ultrasound, cow, portal flow, hepatic flow, portal vein, caudal vena cava

## Abstract

**Simple summary:**

Knowledge of physiological portal and hepatic blood flow in cattle is essential for the use of Doppler ultrasound for diagnostic purposes. In this paper, we describe a protocol for the systematic ultrasonography evaluation of the portal and hepatic system in cattle and report the reference values for healthy cows.

**Abstract:**

In the past, hepatic blood flow in cows was invasively characterized to investigate different pathologies and physiological conditions. However, hepatic blood flow can be easily evaluated with transabdominal Doppler ultrasound. Sixteen healthy adult non-lactating, non-pregnant Holstein-Friesian cows were examined using B-mode and Doppler ultrasound between the right flank and 9^th^ intercostal space to establish the best approach to the different parts of the portal and hepatic vein systems, and determine normal blood flow characteristics. The main portal vein was characterized by a turbulent, high-velocity flow due to the opposing confluence of the splenic and cranial mesenteric veins, while hepatic and caudal vena cava veins have laminar blood flow, in which the phasicity is considered mainly respiratory in origin. Reference values were determined in relation to the anatomical point of observation. In conclusion, transabdominal Doppler ultrasound of the portal system is a simple technique that allows non-invasive characterization of portal and hepatic blood haemodynamics in cows.

## 1. Introduction

B-mode ultrasound of the bovine liver is well described both in physiologically normal and pathological conditions, and has also been used to guide percutaneous sampling and other types of experiments [1,2,3]. Invasive techniques have been used to study hepatic hemodynamics [1,3,4,5,6]. However, more recently, Doppler ultrasound has gained further importance as a non-invasive technique [2,7]. Doppler ultrasound is a non-invasively diagnostic imaging technique that has been widely used in human and veterinary medicine to characterize the blood flow associated with normal and pathological conditions of the liver or as a reflection of systemic conditions in hepatic circulation [8,9,10]. It has been used in cows to characterize the blood flow in different vessels and conditions (i.e., “milk vein” [11], musculophrenic vein in healthy cows [12], dry and lactating periods [13,14], and carotid artery and external jugular vein [15]). Only one report has described the portal vein flow in cases of fatty liver disease [2], and, as far we are aware, no Doppler reference values for portal and hepatic veins have yet been published in the relevant literature.

The aim of this study was to determine the normal features of portal and hepatic blood flow in dairy cattle as a basis for further characterization of different physiological variations and pathologies, and to standardize an ultrasonographic Doppler protocol as in other species.

## 2. Materials and Methods

All experiments performed followed Spanish standards for the protection of animals used for scientific purposes. The procedures applied were supervised by the Bioethics Committee of the Rof-Codina Veterinary Teaching Hospital, University of Santiago de Compostela (Lugo, Spain), and qualified as non-invasive.

### 2.1. Animals

Sixteen healthy non-lactating, non-pregnant adult Holstein-Friesian cows ranging in age from 4.3 to 10.1 years (mean 6.0 years), with a mean weight of 540 kg (range from 470 to 604 kg) were used. Animals received a daily maintenance ration of hay provided *ad libitum* and 2 kg of concentrate (given in two equal rations at about 8:00 and 19:00) throughout the whole study period. All animals had a normal hematological, biochemical, and urine analyses [16]. The cows were held in the installations of the Rof-Codina Veterinary Teaching Hospital, University of Santiago de Compostela, Spain.

### 2.2. B-Mode and Color Doppler Ultrasonography

**Figure 1:**
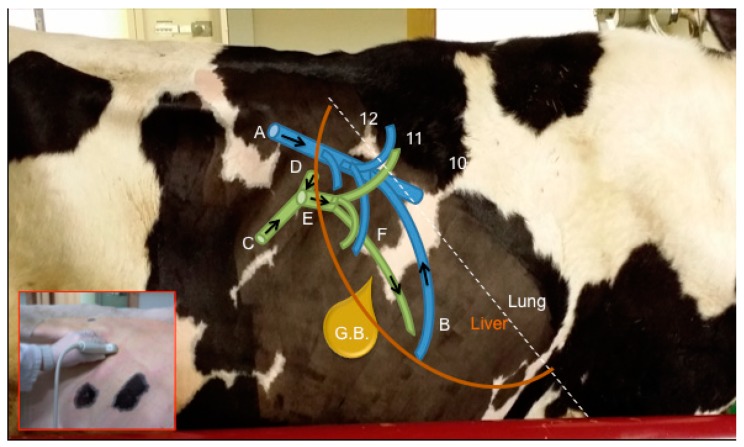
Schematic drawing of the main vessels included in this study. Blue vessels = A—Caudal vena cava, B—Hepatic vessels; Green vessels = C—Cranial mesenteric vein, D—Splenic vein, E—Main Portal vein, F—Portal branches. G.B. = Gall Bladder. Arrows show direction of blood. Numbers indicate specific intercostal spaces.

For this procedure, animals were standing in stocks without sedation. Hair was clipped and the skin washed between the right flank and the 5^th^ right intercostal space (ICS) [1], and between the dorsum and a line crossing from the elbow to the right knee. Ultrasound gel was then applied to ensure good contact between the probe and the skin. A complete B-mode [1], color, and pulsed-wave Doppler ultrasound examination of the portal and hepatic vein systems was performed with a convex array transducer (1–9 MHz) (Esaote MyLab-ClassC^®^, Esaote, Barcelona, Spain) by the same operator (JDBV), between 17:00 and 19:00 (a schematic drawing of the anatomy under investigation is shown in Figure 1). 

For complete characterization of hepatic blood flow, we considered the main portal vein and its tributaries (cranial mesenteric and splenic veins), intrahepatic portal branches, caudal vena cava, and hepatic veins.

For each vessel studied, Doppler measurements were made by aligning the probe as needed to obtain a sagittal image of the vessel with the smallest achievable angle of insonation (always less than 60° for angle correction in Doppler measurements), with a sample volume of 4–9 mm located in the center of the vessel (depending on vessel size) to obtain the blood flow spectrum. The Doppler spectrum was qualitatively evaluated for cardiac and respiratory phasicity by comparing the oscillations in the Doppler trace with the cardiac frequency (obtained by pulse palpation and prior Doppler evaluation of the aorta) and with respiratory motions (visually observed). Mean velocity (Vmean), maximum velocity (Vmax), reverse maximum velocity (Vrev), and blood flow (F) were measured at least 3 times in each vessel for each access point with the built-in software of the ultrasound machine and the maximum velocity method [17] for Vmean and F. In order to calculate the F parameter, the diameter of the vessel was measured in the same image and sagittal plane as the Doppler spectrum.

### 2.3. Statistical Analysis

All statistical analyses were conducted using SPSS for Windows (Version 21, IBM Corp., Armonk, NY, USA). The normality of data distribution was checked using the Kolmogorov-Smirnov test. One-way ANOVA was used to check for possible differences attributable to age and weight. 

## 3. Results and Discussion

### 3.1. General

In all cows, the liver was clearly visible by ultrasonography between the right flank and the right 8^th^ ICS, as previously described [1], except in one cow in which it was only visible from the 11^th^ ICS. None of the cows examined had any B-mode ultrasonographic abnormalities in the liver or adjacent structures. The portal vein and its portal branches were ventrolateral to the hepatic venous system and caudal vena cava, as previously described [1,18]. 

Doppler measurements were made between the right flank and the 9^th^ ICS. No further cranial Doppler signals could be obtained because of the absence of liver parenchyma, small vessel size, or the parenchyma being hidden by the lung. 

The mean velocity can be obtained by different methods [17,19]: directly with the ultrasound software, including the whole vessel diameter within the sample size (uniform insonation method); or by estimation, determining the maximum velocity (by one-off measurement or from a trace, as in our case) of the Doppler spectrum and multiplied by an arithmetic factor of 0.57 to correctly estimate mean velocity [17]. We applied the average maximum velocity method to our measurements to identify whether turbulence was attributable to a disturbed flow or tissue noise (main problem with the uniform insonation method). Different authors have used a non-corrected average maximum velocity profile method, and a correction factor of 0.57 must thus be applied to the values reported [2,7,13,14] for both Vmean and F values (which depend on the first factor). In the maximum velocity method, the Doppler spectrum is measured over a small region (referred to as “sample volume”) in the center of the vessel. However, as a result of respiratory movements, which slightly alter the position of the abdominal structures, the sample is not always in the center of the vessel. This can lead to artifactual turbulence due to tissue motion, as in the insonation method. However, in our experience, tissue motion does not greatly affect the Doppler spectrum obtained, as the vessel diameter is large enough to ensure that the sample remains within the vessel lumen most of the time.

As with venous flow, measurements must be adequate to depict the type of flow (laminar vs. turbulent) and phasicity, if it exists. For this purpose, some authors have used pulsatility indices (venous pulsatility index (VPI)) as described for humans [2,20], under the assumption that the variations encountered in portal flow are cardiac in origin. As we found no significant cardiac influence but a respiratory influence in Doppler waveform, we considered that VPI was not appropriate for characterizing the portal vein Doppler spectrum. We obtained Vmax and Vrev in addition to F and Vmean to characterize the portal and hepatic blood flow, similarly to other authors [7,13,14], and represent differences between laminar and turbulent flow (difference between Vmax, Vrev, and Vmean). 

As age and weight did not influence any of the variables, all cows were included in a single group. The results are presented as arithmetic means, 95% confidence interval (CI), median, minimum, and maximum values. As measurements can vary depending on the access point, we considered those measures taken through the ICS with the highest number of valid observations obtained (most frequent ICS for each vessel) in order to establish reference values.

### 3.2. Portal System

**Figure 2:**
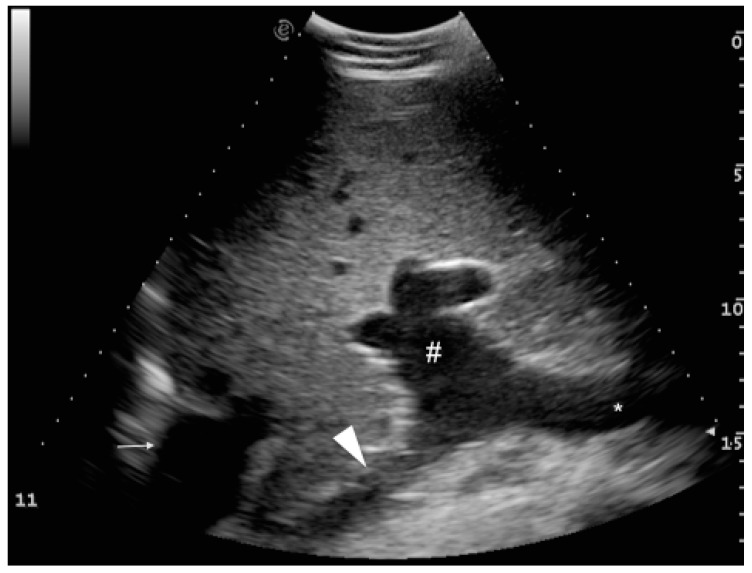
Ultrasound image showing the typical inverted “T” shape of the image due to the confluence of the splenic (arrowhead) and cranial mesenteric veins (*), conforming the main portal vein (#) at the *porta hepatis*. Image obtained in a slightly craniocaudal orientation in the 11^th^ intercostal space (depth is shown on the right of the image). Dorsal location is on the left of the image. Caudal vena cava is located to the left of the image (small arrow).

In order to characterize the portal system, we considered the splenic and cranial mesenteric veins, which are the main vessels conforming the portal vein (not previously described used ultrasound). They have a characteristic typical identifiable inverted T-shape image (Figure 2), which can be useful for identifying these vessels in the ultrasound examination. This image is obtained in an oblique transverse plane, where the splenic vein comes from the medial side of the abdomen joining the cranial mesenteric vein that travels with a caudolateral to craniomedial direction, to conform a short thick main portal vein in an almost perpendicular orientation from the aforementioned two vessels. 

**Figure 3:**
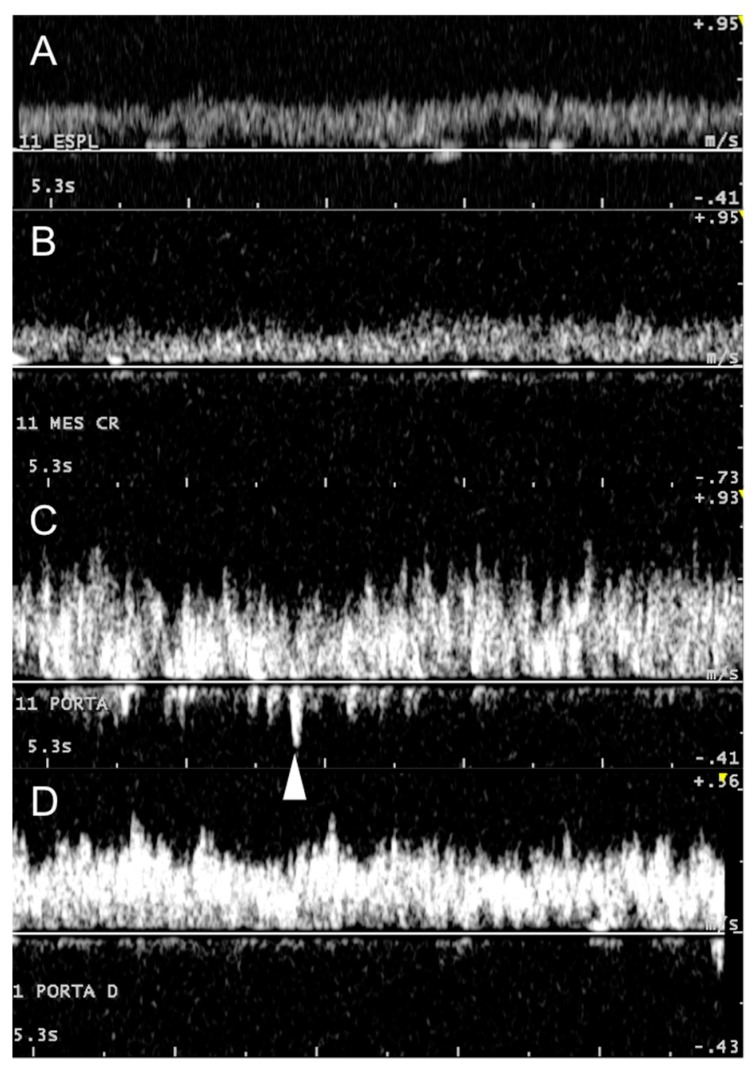
Doppler spectrum of the portal system, from afferent vessels to its main bifurcation within the liver. The images clearly show how portal flow changes from a mainly laminar to a turbulent flow, returning gradually to laminar. (**A**) splenic vein, (**B**) cranial mesenteric vein (Mes Cr), (**C**) main portal vein, and (**D**) right portal branch (Porta D). White arrowhead depicts maximum reverse flow in the main portal vein.

The cranial mesenteric (Table 1) and splenic veins (Table 2) were mainly visualized in the 12^th^ and 11^th^ ICS. Some difficulties may be encountered when trying to align the cranial mesenteric vein appropriately for Doppler examination between the ribs, because of its parallel position in relation to the abdominal wall. The transducer must therefore be angled to obtain adequate insonation angles (<60°). Although the splenic vein always has a suitable angle of insonation, its deep location can sometimes decrease the Doppler signal. These vessels have a typical laminar hepatopetal flow that gradually turns into a turbulent flow when they fuse to form the main portal vein, where the turbulence is maximal (Figure 3).

Considering the values obtained at the most frequent ICS for each vessel, the splenic vein (12^th^ ICS) had higher values of Vmean and Vmax than and similar F and Vrev values to the cranial mesenteric vein (11^th^ ICS), indicating a comparable contribution to the portal blood flow. 

**Figure 4:**
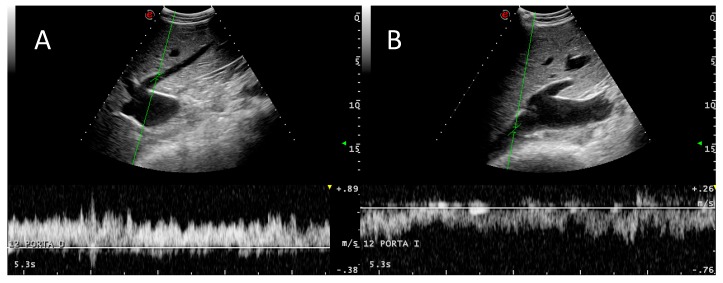
Branching of the main portal vein. (**A**) One of the small branches that emerge from the main trunk and enter the right and middle hepatic lobes. (**B**) The left portal vein is the largest branch that can be easily visualized towards the distal and ventral part of the liver (left lobe). Turbulent flow can be seen in both vessels.

The portal vein and its portal branches could not be observed in the flank ( Table 3; Table 4). The main portal vein can be explored longitudinally in a craniocaudal orientation mostly in the 11^th^ ICS (12/16). As already mentioned, it is sometimes difficult to align the ultrasound probe within the intercostal space for Doppler examination due to a small window for ultrasound recording between the ribs. The portal vein rapidly ramifies in many portal branches of different size that travel in multiple directions, observed cranially to the 12^th^ ICS until the 9^th^ ICS. The right and caudal lobe branches are multiple, variable, and small, emerging directly from the main portal vein or from a single/multiple right trunk and, as lobe separation is not evident in the bovine liver, these branches cannot be confidently labelled by ultrasound examination. This finding is consistent with descriptions in the anatomical literature [18,21] and is referred to occasionally as “stellate branches” [1] (Figure 4A). The largest branch that can be identified travelling deep ventromedially corresponds to the proximal part of the left portal vein or *pars transversa* [18,21] (Figure 4B). 

Regarding blood flow (Table 3 and Table 4), turbulence and F are higher in the main portal vein (characterized by high peak Vmax and high Vrev values) and gradually decrease in the portal branches towards the periphery as the vessels taper, reaching their lowest velocity at the 9^th^ ICS. The results indicate an increase in velocity and flow from the afferent vessels, the splenic and cranial mesenteric veins, to the confluent main portal vein, which distributes blood throughout the liver parenchyma with a consequently gradually slower speed and flow as the portal veins decrease in size. Similarly, turbulence varies from minimum in the splenic and cranial mesenteric veins to maximum in the main portal vein, and then decreases gradually.

Our findings are consistent with those of an invasive experiment [5], in which it was found that the turbulent nature of the portal vein flow was due to a high angle of insertion between splenic and cranial mesenteric veins. We hypothesized that turbulence in the main portal vein will not be a consequence only of the opposite orientation of the splenic and mesenteric veins, but also of a large increase in the diameter of the vein from these to the main portal vein that also generates turbulence in longitudinal vessels, not only in ramifications [19].

**Figure 5:**
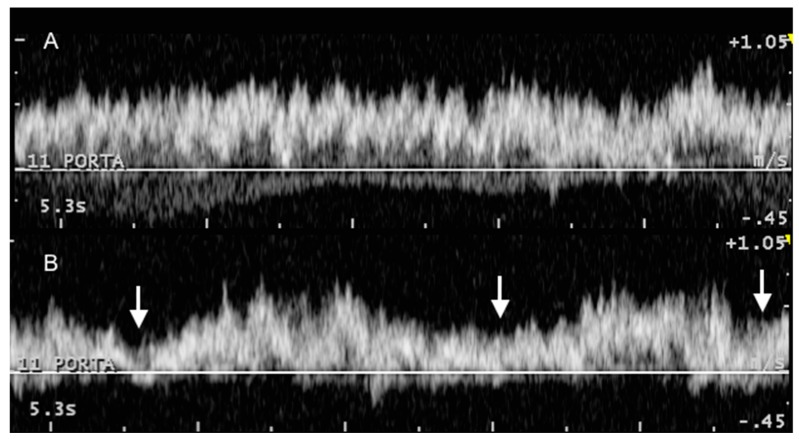
Variation in Doppler phasicity of the portal vein at rest (**A**) and with deep respiratory movements (**B**), obtained consecutively in the same animal and anatomical point (11 intercostal space). White arrows indicate inspiration movements.

Although the portal vein Doppler spectrum is characterized by a marked turbulent hepatopetal flow, some phasicity can be observed and correlated with respiratory movements that consequently change abdominal pressure and portal flow (Figure 5), as seen in dogs [17] and also in cows after invasive examination [5]. 

Starke [2] considered the variations cardiac in origin and, as in this study, no ECG correlation was obtained, as the Doppler spectra could not be directly correlated with cardiac cycle. Only 3 of the 16 cows presented reverse flow in systemic veins (Table 5 and Table 6), showing that pulsatility due to cardiac cycle is much less likely to cause variation in the portal vein blood flow. We believe that if these variations were cardiac in origin, phasicity in the hepatic veins would be expected with synchronous cardiac influence as in humans or dogs; however, this was not observed. Therefore, we should not refer to “pulsatility” of the portal vein, as the flow is not pulsatile, and the variations must be caused by changes in abdominal pressure (i.e., respiratory cycle) and in the amount of blood emitted by the gastrointestinal tract and spleen. Furthermore, based on studies in humans [22], sheep [23], and cattle [2,6,24,25], the portal blood flow depends on posture, physical activity, feed intake, age, and milk yield, which also influence the size and thickness of the liver as body weight does. The correlation between stage of pregnancy and diameter of the portal vein may also influence the portal blood flow [25]. In this study, as all cows were given the same diet and were neither pregnant nor lactating at the time of investigation, these factors were not investigated and must be considered in future research. Body weight and age did not significantly influence the results, which could therefore be taken as reference values to evaluate other factors such as diet, pregnancy, and milk yield. 

### 3.3. Caudal Vena Cava and Hepatic Veins

Hepatic veins and caudal vena cava were best visualized between the flank and the 11^th^ ICS, but could be examined until the 9^th^ ICS for smaller and distal hepatic branches. The best approach to the caudal vena cava (Table 5) was obtained from the right flank, where the ultrasound probe could be best aligned (craniocaudal direction), although it was achievable from the 12^th^ (8/16) and the 11^th^ ICS (4/16). 

**Figure 6:**
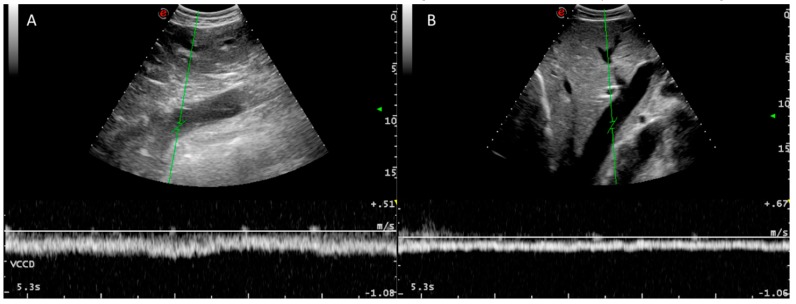
Doppler spectra of the caudal vena cava (**A**) and a hepatic vein (**B**). A typical laminar venous flow can be observed in both cases.

The right and middle hepatic veins and their branches are anatomically variable [18] but consistently visualized with an easy alignment for Doppler investigation because of their lateromedial orientation, which allows insonation angles close to 0° in many instances (Figure 6). 

Hepatic veins are mainly found between the 12^th^ (11/16) and the 10^th^ ICS (12/16), but are also found between the flank (4/16) and the 9^th^ ICS (8/16) in some individuals (Table 6).

The blood flow between the caudal vena cava and its hepatic veins had a laminar Doppler profile without reverse flow (Figure 6), with variations due to respiratory changes in abdominal pressure without cardiac phasicity as in the portal system, except for some measurements in three cows (Table 5 and Table 6). Vrev was inconsistently detected in three animals. Reverse flow was inconsistently found and we hypothesized that it could be caused by excessive abdominal pressure due to respiration effort. These three animals had normal levels of the other individual portal system Doppler values, indicating that cardiac influence is not transmitted to the portal system by the hepatic sinusoids. In healthy dogs and humans, reverse flow in the caudal vena cava is correlated with atrial contraction during atrial systole [10,19]. We did not detect any cardiac pathology in these individuals by auscultation or basic echocardiography. ECG correlation with the Doppler spectrum could explain the cardiac influence on the caudal vena cava and hepatic veins in the three cows in which Vrev was detected. 

The main difficulty in obtaining Doppler measurements in the caudal vena cava was the caudocranial orientation of this vessel. Fortunately, a flank approach provided easy access with acceptable Doppler angles. However, a high variability in the diameter of the caudal vena cava diameter was observed because of its triangular shape within the liver in cross-section. In order to calculate F, the vessel diameter is measured in the sagittal plane (to ensure vessel orientation and Doppler angle), where it cannot be confirmed if this measurement correctly estimates vessel area, as the diameter is transformed into area assuming that it is basically round. We recommend that an additional transverse image be obtained in future studies to measure the caudal vena cava with a trace tool in cross-section. Although this would extend the time of ultrasound examination, more accurate results would be obtained. As this method may also overestimate the vessel area due to an oblique angle of approach (form round to oval), care must be taken according to the method applied.

As in portal branches, all parameters in hepatic veins depend on vessel diameter and distance from the caudal vena cava, related to peripheral tapering of these vessels. An anecdotally similar Vmean between the intrahepatic portal and hepatic vessels can be observed, reflecting some correspondence in liver tension/liver sinusoids.

## 4. Conclusions

Reference values for portal and hepatic blood flow in adult healthy cattle were obtained using Doppler ultrasonography as a non-invasive reliable method, reflecting the residual influence of cardiac phasicity in both venous systems and a characteristic turbulent portal blood flow. Further research must be conducted to achieve a better understanding of the factors influencing hepatic circulation in cows (e.g., influence of ruminal movements, pregnancy, diet, and other factors).

## Figures and Tables

**Table 1:** Results of ultrasonography examination of the cranial mesenteric vein expressed as mean (95% confidence interval) and median (minimum-maximum). Values obtained in the most frequent location are highlighted in bold.

Location		Flank	12 ICS	11 ICS	10 ICS	9 ICS
No. of observations (no. cows)						
General		10 (3)	**34 (8)**	**30 (7)**	3 (1)	NA
Reverse maximum velocity		4 (1)	**17 (5)**	**17 (6)**	3 (1)	NA
Mean velocity (cm/s)						
Mean (95% CI)		15.0 (12.8–17.3)	**16.0 (15.0**–**17.1)**	**13.8 (13.1**–**14.6)**	17.5 (7.2–27.7)	NA
Median (min-max)		13.7 (11.4–20.5)	**15.7 (10.8**–**21.7)**	**14.2 (9.7**–**17.1)**	15.4 (14.8–22.2)	
Maximum velocity (cm/s)						
Mean (95% CI)		37.8 (32.9–42.7)	**39.1 (35.7–42.4)**	**34.3 (32.4–36.3)**	41.0 (10.8–71.2)	NA
Median (min–max)		37.0 (29.0–51.0)	**36.0 (28.0–60.0)**	**34.5 (24.0–47.0)**	35.0 (33.0–55.0)	
Reverse maximum velocity (cm/s)						
Mean (95% CI)		23.0 (9.8–36.2)	**19.9 (16.1–23.7)**	**16 (13–19)**	21.7 (16.5–26.8)	NA
Median (min–max)		25.5 (11.0–30.0)	**19.0 (9.0–34.0)**	**17.0 (8.0–26.0)**	21.0 (20.0-24.0)	
Blood flow (F)						
L/min	Mean (95% CI)	3.44 (2.37–4.51)	**3.93 (3.37–4.48)**	**2.98 (2.66–3.31)**	4.35 (3.05–5.65)	NA
	Median (min–max)	2.78 (1.08–6.01)	**3.57 (1.93–7.78)**	**3.14 (1.01–4.06)**	4.19 (3.92–4.93)	NA
ml/min/kg	Mean (95% CI)	6.88 (4.41–9.35)	**7.42 (6.5–8.34)**	**5.86 (5.11–6.61)**	8.14 (5.71–10.6)	NA
	Median (min–max)	5.2 (3.25–12.8)	**7.4 (3.63–13.1)**	**6.05 (1.81–9.16)**	7.85 (7.34–9.24)	NA

ICS: intercostal space; NA: not accessible; CI: confidence interval.

**Table 2:** Results of ultrasonography examination of the splenic vein expressed as mean (95% CI) and median (minimum–maximum). Values obtained in the most frequent location are highlighted in bold.

Location		Flank	12 ICS	11 ICS	10 ICS	9 ICS
No. of observations (no. cows)						
General		3 (1)	**35 (10)**	**40 (12)**	9 (3)	NA
Reverse maximum velocity		3 (1)	**19 (6)**	**8 (3)**	2 (1)	NA
Mean velocity (cm/s)						
Mean (95% CI)		27.2 (12.2–42.2)	**24 (22.5–25.4)**	**24 (22.7–25.2)**	20 (18.5–21.5)	NA
Median (min–max)		26.2 (21.7–33.6)	**23.9 (17.7–33.9)**	**23.4 (16.5–31.9)**	19.9 (17.7–23.4)	NA
Maximum velocity (cm/s)						
Mean (95% CI)		68.3 (36.4–99.9)	**56.8 (53.3–60.2)**	**55.1 (52.5–57.7)**	47 (42.5–51.5)	NA
Median (min–max)		63 (59–83)	**56 (39–79)**	**55 (41–78)**	45 (42–57)	NA
Reverse maximum velocity (cm/s)						
Mean (95% CI)		19.3 (9.9–28.7)	**19.2 (16.1–22.3)**	**19.4 (15.6–23.2)**	13.5 (7.1–19.8)	NA
Median (min–max)		21.0 (15.0–22.0)	**18.0 (10.0–31.0)**	**18.5 (13.0–26.0)**	13.5 (13.0–14.0)	NA
Blood flow (F)						
L/min	Mean (95% CI)	4.17 (1.42–6.92)	**3.95 (3.48–4.43)**	**3.58 (3.04–4.13)**	2.37 (1.94–2.8)	NA
	Median (min–max)	3.63 (3.44–5.44)	**3.73 (1.71–7.44)**	**3.17 (1.78–9.62)**	2.24 (2–3.78)	NA
ml/min/kg	Mean (95% CI)	7.99 (2.71–13.3)	**7.5 (6.56–8.43)**	**6.74 (5.82–7.67)**	4.3 (3.65–4.95)	NA
	Median (min–max)	6.95 (6.58–10.4)	**7.03 (3.37–13.4)**	**5.62 (3.5–16.2)**	4.07 (3.38–6.36)	NA

ICS: intercostal space; NA: not accessible; CI: confidence interval.

**Table 3:** Results of ultrasonography examination of the portal vein expressed as mean (95% CI) and median (minimum–maximum). Values obtained in the most frequent location are highlighted in bold.

Location		Flank	12 ICS	11 ICS	10 ICS	9 ICS
N° of observations (n° cows)						
General		NA	16 (4)	**48 (12)**	25 (7)	NA
Reverse maximum velocity		NA	16 (4)	**48 (12)**	21 (6)	NA
Mean velocity (cm/s)						
Mean (95% CI)		NA	26.6 (22.4–30.8)	**23.4 (21.8–25)**	25.5 (23–28)	NA
Median (min–max)		NA	23.9 (17.1–39.9)	**24.2 (10.8–33.1)**	23.9 (16.5–41.6)	NA
Maximum velocity (cm/s)						
Mean (95% CI)		NA	71.7 (59.2–84.3)	**64.8 (60.3–69.2)**	65.3 (58.9–71.7)	NA
Median (min–max)		NA	60 (46–111)	**68.5 (29–93)**	62 (46–113)	NA
Reverse maximum velocity (cm/s)						
Mean (95% IC)		NA	38.2 (31.5–44.9)	**24.9 (22.2–27.5)**	22.5 (17.5–27.4)	NA
Median (min–max)		NA	41 (16–57)	**25.5 (8–43)**	20 (9–54)	NA
Blood flow (F)						
L/min	Mean (95% CI)	NA	13.3 (9.75–16.9)	**13.1 (11.7–14.5)**	12.4 (11.5–13.3)	NA
	Median (min–max)	NA	12.2 (3.78–23.6)	**13.5 (3.04–21.0)**	12.1 (9.31–17.0)	NA
ml/min/kg	Mean (95% CI)	NA	26.5 (18.9–34.2)	**25.5 (23–28.1)**	22.5 (21– 23.9)	NA
	Median (min–max)	NA	23.1 (8.05–50.3)	**27 (5.82–40.8)**	21.8 (16.4–29.3)	NA

ICS: intercostal space; NA: not accessible; CI: confidence interval.

**Table 4:** Results of ultrasonography examination of the intrahepatic portal veins expressed as mean (95% CI) and median (minimum–maximum). Values obtained in the most frequent location are highlighted in bold.

Location		Flank	12 ICS	11 ICS	10 ICS	9 ICS
N° of observations (n° cows)						
General		NA	43 (9)	**77 (15)**	43 (12)	15 (4)
Reverse maximum velocity		NA	30 (7)	**55 (14)**	29 (9)	10 (2)
Mean velocity (cm/s)						
Mean (95% CI)		NA	16.4 (15.0–17.8)	**18.3 (17.3–19.2)**	16 (14.5–17.5)	13.1 (10.5–15.7)
Median (min–max)		NA	16.0 (9.1–29.1)	**17.7 (8.0–28.5)**	16.0 (5.1–25.9)	10.3 (8.0–20.5)
Maximum velocity (cm/s)						
Mean (95% CI)		NA	44.5 (40.3–48.8)	**50.9 (47.4–54.4)**	42.3 (38.6–45.9)	34.3 (29.8–38.8)
Median (min–max)		NA	42 (26–80)	**46 (30–94)**	42 (12–67.6)	33 (20.2–46)
Reverse maximum velocity (cm/s)						
Mean (95% CI)		NA	22.2 (19.3–25)	**21.6 (19.2–24)**	18.1 (16.1–20.1)	18.2 (15.1–21.3)
Median (min–max)		NA	24 (8–37)	**19.2 (9–46)**	19 (6–29)	18 (11–26)
Blood flow (F)						
L/min	Mean (95% CI)	NA	1.94 (1.47–2.4)	**2.63 (2.25–3)**	2.02 (1.52–2.51)	1.26 (0.92–1.6)
	Median (min–max)	NA	1.84 (0.07–6.69)	**2.31 (0.22–9.44)**	1.79 (0.04–6.99)	1.18 (0.47–2.62)
ml/min/kg	Mean (95% CI)	NA	3.81 (2.84–4.77)	**4.89 (4.22–5.57)**	3.87 (2.97–4.77)	2.65 (1.79–3.51)
	Median (min–max)	NA	3.62 (0.14–14.2)	**4.26 (0.42–16.3)**	3.68 (0.07–12)	2.47 (0.81–5.97)

ICS: intercostal space; NA: not accessible; CI: confidence interval.

**Table 5:** Results of ultrasonography examination of the caudal vena cava expressed as mean (95% CI) and median (minimum–maximum). Values obtained in the most frequent location are highlighted in bold.

Location		Flank	12 ICS	11 ICS	10 ICS	9 ICS
No. of observations (no. cows)						
General		**56 (11)**	29 (8)	16 (4)	NA	NA
Reverse maximum velocity		**12 (3)**	0 (0)	0 (0)	NA	NA
Mean velocity (cm/s)						
Mean (95% CI)		**19.9 (17.9–21.8)**	18.1 (16.6–19.7)	16.5 (13.7–19.2)	NA	NA
Median (min–max)		**18.6 (6.8–42.2)**	18.2 (9.1–24.4)	15 (10–27)	NA	NA
Maximum velocity (cm/s)						
Mean (95% CI)		**50.3 (45.8–54.9)**	42.6 (39.3–46)	39 (33.4–44.6)	NA	NA
Median (min–max)		**49 (18–110)**	43 (17–57)	37 (23–61)	NA	NA
Reverse maximum velocity (cm/s)						
Mean (95% CI)		**17.9 (13.1–22.7)**	*	*	NA	NA
Median (min–max)		**14.5 (8–30)**	*	*	NA	NA
Blood flow (F)						
L/min	Mean (95% CI)	**4.58 (3.83–5.33)**	6.09 (5.24–6.93)	3.94 (2.76–5.12)	NA	NA
	Median (min–max)	**4.33 (0.47–12.40)**	5.51 (1.77–10.40)	3.04 (1.60–8.81)	NA	NA
ml/min/kg	Mean (95% CI)	**9.17 (7.51–10.80)**	11.30 (9.71– 12.80)	7.17 (4.93– 9.41)	NA	NA
	Median (min–max)	**8.38 (1.08–30.10)**	10.90 (3.05– 19.20)	5.52 (2.85– 16.90)	NA	NA

ICS: intercostal space; NA: not accessible. * No reverse velocity observed.

**Table 6:** Results of ultrasonography examination of the hepatic veins expressed as mean (95% CI) and median (minimum–maximum). Values obtained in the most frequent location are highlighted in bold.

Location		Flank	12 ICS	11 ICS	10 ICS	9 ICS
No. of observations (no. cows)						
General		12 (4)	**52 (11)**	**43 (12)**	**42 (11)**	26 (8)
Reverse maximum velocity		3 (1)	**2 (1)**	**4 (1)**	**2 (1)**	4 (1)
Mean velocity (cm/s)						
Mean (95% CI)		12.9 (9.9–16.0)	**14.9 (13.7–16.2)**	**12.8 (11.9–13.8)**	**8.83 (7.98–9.69)**	9.27 (8.33–10.2)
Median (min–max)		11.7 (7.4–21.7)	**13.7 (7.4–26.8)**	**12.5 (7.4–19.4)**	**8.55 (5.1–17.1)**	9.4 (5.7–13.9)
Maximum velocity (cm/s)						
Mean (95% CI)		31.4 (24.0–38.8)	**35.2 (32.1–38.2)**	**31.1 (28.7–33.5)**	**21.9 (19.8–24)**	23.3 (20.9–25.6)
Median (min–max)		35 (18–53)	**33 (19–71)**	**31 (18–52)**	**21 (11–38)**	23 (15–36)
Reverse maximum velocity (cm/s)						
Mean (95% CI)		17.7 (8.94–26.4)	**25.5 (19.1–31.8)**	**19 (8.69–29.3)**	**11 (–1.7–23.7)**	7 (4.75–9.25)
Median (min–max)		18 (14–21)	**26 (25–26)**	**21 (10–25)**	**11 (10–12)**	7 (5–8)
Blood flow (F)						
L/min	Mean (95% CI)	1.84 (0.77–2.92)	**2.28 (1.86–2.7)**	**1.35 (1.03–1.67)**	**0.76 (0.63–0.9)**	0.95 (0.69–1.22)
	Median (min–max)	1.03 (0.19–4.92)	**1.73 (0.32–5.38)**	**0.81 (0.3–3.71)**	**0.62 (0.19–1.61)**	0.8 (0.09–2.72)
ml/min/kg	Mean (95% CI)	4.13 (1.51–6.74)	**4.43 (3.58–5.28)**	**2.65 (1.94–3.35)**	**1.47 (1.2–1.73)**	1.86 (1.27–2.45)
	Median (min–max)	2.2 (0.32–11.9)	**3.61 (0.55–10.8)**	**1.6 (0.56–8.98)**	**1.21 (0.35–3.68)**	1.47 (0.14–6.22)

ICS: intercostal space; NA: not accessible.
